# Progress of Veterinary Science

**Published:** 1887-01

**Authors:** 


### PROGRESS OF VETERINARY SCIENCE.

Iron-weed as a Cause of Abortion.—Some years ago, a physician living in the lower part of our city, submitted to me for determination a root fragment which he said was used in the circle of his practice in a rural district as a domestic emenagogue. Not recognizing the root, he afterwards brought to me a portion of the stem and leaves of a plant which proved to belong to the common iron-weed, *vernonia noveboracensis*, growing abundantly in the meadows. It has occurred to me that if this plant has the quality attributed to it, that when cut among hay and fed to cattle, it may be one of the causes of abortion among cows.

Joseph Leidy.

Medicine as Practiced by Animals.—Wm. O'Neill, M. D., of Lincoln, in England, relates the following in a letter addressed to the *British Medical Journal* dated February 15th, 1883.

A young cat which was born blind, and which shows extraordinary acts of instinct, amounting almost to reasoning intelligence, began to suffer from epileptiform fits, and abscesses of the face in the summer of the past year. From the age of three or four months, the animal could go all over the house and garden nearly as well as if she had her sight. So long as pieces of furniture were in their usual places, she rarely, even in her playful gambols, knocked herself against any of them; but several times, when chairs and tables were displaced, she has hit her head with great force against them, and on one occasion her head came into violent collision with a mowing machine. The fits and abscesses were, therefore, sufficiently accounted for by the injuries which the cat received. An abscess, which recurred every ten or twelve days, formed on the right cheek, and usually opened under the right eye and into the nostrils. At the outset, the matter discharged was pus, but afterwards blood escaped with the pus, and sometimes blood, without any appreciable quantity of pus, escaped by the same openings. The fits, which gradually increased in violence and frequency, were not much under the control of bromide of potassium.

The great amiability of temper the poor animal displayed during her sufferings, which occasionally must have been very severe, was remarkable. She could hardly be induced to take any food with the exception of a little cream; consequently she soon wasted away to mere skin and bone. The bowels were generally constipated for days at a time, and her breath had a most unbearable fœtid odor.

I may mention, too, that the poor animal became intensely dropsical, the abdomen becoming distended almost like an inflated bladder. Her heart could be seen to beat violently on her making the slightest exertion, and on several occasions she coughed up quantities of blood. She breathed through the open mouth, owing to the nostrils being blocked up with tenacious matter, which she from time to time endeavored to discharged by violent sneezings.

The animal's treatment of herself was very simple, but as far as it went was, I think, fairly good. She lay in a basket made warm and comfortable, which she rarely left, excepting to crawl to the fireplace in order to procure well-burned cinders, which she ate with avidity. This being observed, willow charcoal was procured, and daily put in a place accessible to her. At first she rather disliked the willow charcoal, but soon she became fond of it. To milk she seemed to have a great repugnance; nevertheless, milk was the means she used to act on her bowels. For days the bowels would not act; then all at once she would begin to lap milk in quantities, which fluid never failed to operate on her bowels in less than an hour. The disliked milk then was the means the cat used to keep the bowels open, and the charcoal was the remedy which she employed in order, in all probability, to correct the fœtid state of the breath, and the emanations from the decomposing secretions of the mouth and nose, and perhaps also to allay some uneasy sensations in her stomach. When the disease had been going on for two or three months, the idea of giving her sulphide of calcium, for the recurrent suppuration, struck me. She was therefore given about a quarter of a grain of the medicine three or four times a day. On the suppuration the calcium acted slowly and surely ; but on the dropsy, which had existed for some months, it acted like a charm, for in a few weeks the water disappeared without leaving a trace. In fact, all the formidable symptoms rapidly passed away, the suppuration being the last to disappear, owing doubtless to some disease of the bones of the face.

The cat is now a beautiful animal, fat and with a fine white fur, and as playful as a kitten. It had been often said by those who saw the animal when in the height of her illness, that it was cruelty not to destroy her. Her recovery is, however, most instructive, not only on account of the means which she herself used, but more especially on account of the favorable effects the sulphide of calcium had on the dropsy.

---

### THE ROYAL (DICK) VETERINARY COLLEGE.

Owing to the steady increase in the number of students attending this institution during recent years, and to the extension and development of the methods of teaching, both hospital and class-room accommodation has gradually become inadequate. Three years ago the trustees reconstructed a section of the College property, which had previously been let as dwelling-houses, and converted it into class-rooms. This afforded a temporary relief, but it soon became evident that nothing short of a complete reconstruction of the then existing premises would suffice to provide the requisite accommodation. The plan of the remodelled premises was prepared from an outline of the teaching requirements supplied by the professors of the College. It is intended that the new premises will, in respect of completeness and adaptation for teaching, occupy a foremost place among similar institutions. The main entrance to the new buildings is, as before, in the form of an archway, opening off Clyde Street. The ground floor to the left of the entrance comprises the Principal's office and waiting-rooms, while to the right of the entrance a house is provided for the janitor. The second story on this site consists of the museum, which communicates with the pathological department at its west extremity, and with the anatomical rooms on the east. Over the museum are placed pathological and physiological laboratories and class-rooms for practical chemistry and practical microscope work, in connection with the classes of physiology and pathology. On the east side the ground floor is occupied mainly by stalls and loose boxes, but it comprises also a pharmacy room and an operation room. The remaining buildings on this side are devoted to the anatomical department, and comprise the dissecting room, a lecture theater, macerating room, professors' private work room, etc. The dissecting room is a very spacious apartment, after the design of the new dissecting room in the Edinburgh University. It is lit entirely from the north and is provided along one side with a gallery for the display of preserved preparations. A hoist will bring up the dissection subjects from the ground floor, and mechanical appliances will be provided for transporting them with ease to any part of the room. The floor of this room is of asphalt, and it is intended to coat the walls with enamel paint. The anatomical lecture theater is seated for 150 students, and is in communication on either side with the dissecting room and with the museum. Entire subjects can thus be brought in from the dissecting room in order to illustrate the lectures and for the same purpose there will be every facility for utilizing preserved preparations from the museum. The buildings on the north extremity comprise on the ground floor a shoeing forge, and hospital accommodation in the shape of dog kennels, stalls and loose boxes. Over this, and forming the second story, are a library and a gymnasium for the use of students; while the upper story on this side forms part of a suite of rooms devoted to the chemical department. On the west side the entire ground floor is occupied by stalls and loose boxes, and over these are placed part of the suite of chemical rooms, a general lecture theater, seated for 200 students, professors' private rooms, etc. The central court, from which access is obtained by staircases to the various departments, is to be entirely roofed with glass and a gallery will extend along one side of the court at the level of the first floor. The staircase at the north end of the court, which fronts the main entrance, will have the lower part in the form of an arcade, in the central niche of which will be placed the memorial statue of Professor Dick, the founder of the institution.

---

PHARMACEUTICALS FOR VETERINARY PRACTICE.—During the recent meeting of the Veterinary and Sanitary Association at Chicago, a very handsome display of preparations especially adapted for veterinary practice was presented by Parke, Davis & Co. It is significant of the progress that veterinary medicine is making, that such large purveyors of drugs should establish especial facilities for supplying the demands of veterinarians, not only the more commonly used preparations in convenient form, but the practitioner's special prescriptions in gelatine-coated balls which are compact and durable.

---

### MECHANICAL HORSE-SHOEING.

The progress and improvements that have taken place during the past few years in the method of treating horses, with a view to the cure and amelioration of the various ailments with which they are affected, have been commensurately equal in importance to the innovations and advancements made in the medical and surgical treatment of human beings.

One of the most important is the patent hoof expander, invented by Dr. Roberge, and for which the Judges at the last American Institute Fair gave him the high honor of the Special Medal of Silver, which is only given, according to the constitution of the institute, to productions of rare merit and extraordinary usefulness.

